# Sequence and Structure Signatures of Cancer Mutation Hotspots in Protein Kinases

**DOI:** 10.1371/journal.pone.0007485

**Published:** 2009-10-16

**Authors:** Anshuman Dixit, Lin Yi, Ragul Gowthaman, Ali Torkamani, Nicholas J. Schork, Gennady M. Verkhivker

**Affiliations:** 1 Graduate Program for Bioinformatics, Center for Bioinformatics, The University of Kansas, Lawrence, Kansas, United States of America; 2 Department of Pharmaceutical Chemistry, School of Pharmacy, The University of Kansas, Lawrence, Kansas, United States of America; 3 Scripps Genomic Medicine, Department of Molecular and Experimental Medicine, Scripps Health and The Scripps Research Institute, La Jolla, California, United States of America; 4 Department of Pharmacology, University of California San Diego, La Jolla, California, United States of America; Keio University, Japan

## Abstract

Protein kinases are the most common protein domains implicated in cancer, where somatically acquired mutations are known to be functionally linked to a variety of cancers. Resequencing studies of protein kinase coding regions have emphasized the importance of sequence and structure determinants of cancer-causing kinase mutations in understanding of the mutation-dependent activation process. We have developed an integrated bioinformatics resource, which consolidated and mapped all currently available information on genetic modifications in protein kinase genes with sequence, structure and functional data. The integration of diverse data types provided a convenient framework for kinome-wide study of sequence-based and structure-based signatures of cancer mutations. The database-driven analysis has revealed a differential enrichment of SNPs categories in functional regions of the kinase domain, demonstrating that a significant number of cancer mutations could fall at structurally equivalent positions (mutational hotspots) within the catalytic core. We have also found that structurally conserved mutational hotspots can be shared by multiple kinase genes and are often enriched by cancer driver mutations with high oncogenic activity. Structural modeling and energetic analysis of the mutational hotspots have suggested a common molecular mechanism of kinase activation by cancer mutations, and have allowed to reconcile the experimental data. According to a proposed mechanism, structural effect of kinase mutations with a high oncogenic potential may manifest in a significant destabilization of the autoinhibited kinase form, which is likely to drive tumorigenesis at some level. Structure-based functional annotation and prediction of cancer mutation effects in protein kinases can facilitate an understanding of the mutation-dependent activation process and inform experimental studies exploring molecular pathology of tumorigenesis.

## Introduction

A central goal of cancer research involves the discovery and functional characterization of the mutated genes that drive tumorigenesis [**1**]. The Human Genome Project has provided researchers with unprecedented insights into the structure and organization of genes. Large-scale resequencing and polymorphism characterization studies have subsequently focused on the identification and cataloguing of naturally occurring gene and sequence variation [**2]**–[**5**]. The Cancer Genome Atlas and related DNA sequencing initiatives have specifically investigated the genetic determinants of cancer [**6**]. These studies have determined that only a fraction of genetic alterations contributing to tumorigenesis may be inherited, while somatically acquired mutations can contribute decisively during the progression of a normal cell to a cancer cell. Protein kinases play a critical role in cell signaling and have emerged as the most common protein domains that are implicated in cancer [**7**]–[**11**]. Although the kinase catalytic domain is highly conserved, protein kinase crystal structures have revealed considerable structural differences between closely related active and highly specific inactive forms of kinases [**12**]–[**17**]. Evolutionary conservation and conformational plasticity of the kinase catalytic domain allow for a dynamic equilibrium between active and inactive kinase forms, which can facilitate regulation of the catalytic activity [**15**]–[**17**]. There are more than 500 protein kinases encoded in the human genome and many members of this family are prominent therapeutic targets for combating diseases caused by abnormalities in signal transduction pathways, especially various forms of cancer [**18**]–[**22**].

The complete sequencing of the human genome and high-throughput generation of genomic data have opened up avenues for a systematic approach to understanding the complex biology of cancer and clinical targeting of activated oncogenes. Large-scale tumor sequencing studies have identified a rich source of naturally occurring mutations in the protein kinase genes with many being simple single nucleotide polymorphisms (SNPs) [**23**]–[**32**]. A subset of these SNPs could occur in the coding regions (cSNPs) and lead to the same polypeptide sequence (synonymous SNPs, sSNPs) or result in a change in the encoded amino acid sequence (nonsynonymous coding SNP, nsSNPs). Resequencing studies of the kinase coding regions in tumors have classified tumor-associated somatic mutations revealing that only a small number of kinase mutations may contribute to tumor formation (known as cancer driver mutations) while the majority could be neutral mutational byproducts of somatic cell replication (known as passenger mutations) [**23**]–[**28**]. While protein kinases have a prominent role in tumorigenesis, commonly mutated protein kinases in cancer appeared to be the exception to the rule and most of kinase driver mutations are expected to be distributed across many protein kinase genes [**27**]. Cancer mutations in protein kinases could often exemplify the phenomenon of oncogene addiction whereby, despite the accrual of numerous genetic alterations over the maturation of a tumor, cancer cells could remain reliant upon particular oncogenic pathways and may become addicted to the continued activity of specific activated oncogenes [**33**], [**34**]. The dominant oncogenes that confer the oncogene addiction effect include ABL, EGFR, VEGFR, BRAF, FLT3, RET, and MET kinase genes [**34**].

The recent discovery of lung cancer mutations in the EGFR kinase domain [**35**]–[**37**] and their differential sensitivity to EGFR inhibitors have suggested that genetic alterations may be associated with structural changes, rendering tumors sensitive to selective inhibitors. Structural determinations of the EGFR [**38**]–[**41**] and ABL cancer mutants [**42**], [**43**] have suggested that molecular mechanisms of kinase activation by cancer mutations and activity signatures of cancer drugs may be associated with the dynamics of functional transitions between inactive and active kinase forms. Biophysical modeling of protein kinase structure and dynamics has revealed important mechanistic features of kinase activation at atomic resolution. Molecular dynamics (MD) simulations of large-scale conformational transitions have been performed for many therapeutically important protein kinases, including HCK kinase [**44**], adenylate kinase [**45**], Src kinase [**46]**–[**51**], cyclin-dependent kinase 5 (CDK5) [**52**], ABL kinase [**53**], KIT kinase [**54**] EGFR, RET and MET kinase domains [**55]**–[**57**]. These studies have suggested that cancer mutations can have a subtle, yet profoundly important functional affect not only on local conformational changes at the mutational site, but also on allosteric regulation and cooperative interactions in signal transduction networks [**58]**, [**59**]. According to the proposed mechanism of kinase activation, structural effect of cancer mutations could manifest in shifting the dynamic equilibrium between inactive and active kinase forms towards a constitutively active kinase, thereby causing deleterious consequences for kinase regulation.

Cancer biology studies of protein kinase genes have integrated genetic, structural and functional approaches to characterize underlying molecular signatures of cancer mutations. High-throughput DNA sequence analysis and functional assessment of candidate cancer mutations in the tyrosine kinase genes have identified point mutations in the conserved hot spots from the activation loop in leukemia-associated tyrosine kinases [**60]**–[**63**]. A high-throughput platform has been used to interrogate the entire FLT3 coding sequence in AML patients and experimentally test the functional consequences of each candidate tumorigenic allele [**63**]. These studies have indicated that rare driver variants could often occur at frequencies indistinguishable from passenger mutations. As a result, functional analysis of candidate mutations identified in genome-wide screens can be ultimately required to determine which mutations contribute to cell transformation. Computational approaches, when combined with structural and functional studies, have also facilitated the identification and prediction of candidate cancer genes and individual alleles contributing to tumorigenesis [**64]**–[**67**].

Bioinformatics tools were recently developed to distinguish between driver and passenger nsSNPs [**68]**, [**69**]. Though quite powerful, generalized prediction methods may fail to achieve the sensitivity and specificity attainable by prediction models tailored to individual protein families. We have developed kinase-targeted machine learning models that focused on nsSNPs in protein kinases by leveraging known sequence-based and structure-based protein kinase features to identify patterns in residues and sequence motifs harboring functionally relevant variations [**70]**–[**72**]. The developed support-vector machine (SVM) method has been shown to differentiate between disease-associated nsSNPs and neutral nsSNPs with ∼80% accuracy [**70**]. These findings have suggested that the predictive power of machine learning models in assessing functionally important mutations can be significantly enhanced by selecting informative attributes characteristic of a specific protein family. Furthermore, we have found that kinase regions harboring a large number of cancer mutations in multiple protein kinases could contain a high proportion of the predicted driver mutations, while kinase subdomains devoid of cancer mutations were more likely to contain passenger mutations [**71]**, [**72**]. These results have suggested that biological characteristics and functional consequences separating cancer driver mutations from passenger mutations in protein kinases may differ from those separating disease-associated from neutral nsSNPs across the entire genome.

The growing body of genetic, molecular and functional information about protein kinases genes, combined with their prominent role as therapeutic targets for cancer intervention have produced an unprecedented explosion of diverse data. A large amount of information about genetic modifications in protein kinase families has been accumulated in different sources, including PupaSNP [**73**], dbSNP database [**74**], Online Mendelian Inheritance in Man (OMIM) from National Center for Biotechnology Information (NCBI) [**75]**, [76****], KinMutBase [**77]**, [**78**], BTKbase [**79**], Human gene mutation database (HGMD) [**80]**, [**81**], Catalogue of Somatic Mutations in Cancer database (COSMIC) [**82**], Protein Kinase Resource (PKR) [**83**], and Mutations of Kinases in Cancer (MoKCa) [**84**]. While current databases and information portals have accumulated a large amount of information on kinase SNPs, there is a growing need for integration and comprehensive mapping of diverse data categories on protein kinase genes within a central resource.

In this work, we introduce Composite Kinase Mutation Database (CKMD), a single repository and integrated bioinformatics resource that consolidated and unequivocally mapped all currently available information on genetic variations in protein kinase genes with sequence, structural and functional data. CKMD and web-based resource are freely available at http://verklab.bioinformatics.ku.edu/database/. The functionality and capabilities of CKMD portal can allow for robust functional annotation of protein kinase genes and enable kinome-wide prediction and structure-functional analysis of cancer mutations. The database-driven analysis of sequence and structure-based signatures of kinase SNPs has clarified salient aspects of sequence conservation patterns and structural profiles of cancer-causing mutations, including the emergence of structurally conserved tumorigenic hotspots across multiple protein kinases. Furthermore, structural modeling and energetic analysis of kinase cancer mutations, which constitute the largest mutational hotspot, have provided useful insights into a common mechanism of kinase activation.

## Results

### Sequence-Structure Classification and Mapping of Kinase SNPs

**Figure 1:**
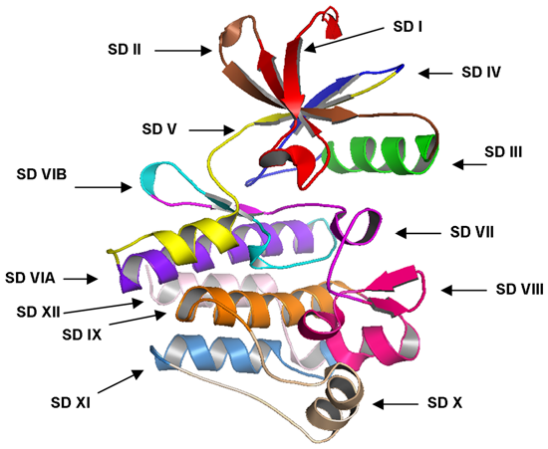
Functional Subdomains of the Kinase Catalytic Core. The kinase catalytic domain was subdivided into 12 subdomains (SD) using the ABL kinase crystal structure (pdb entry 1IEP) as the reference for defining the residue ranges as follows : SD I:242–261(P-loop region); SD2 :262–278; SD3:279–291(αC-helix); SD4:292–309; SD5:310–335 (hinge region); SD6A:336–356; SD6B357–374 (catalytic loop); SD7:375–393 (activation loop) ; SD8:394–416 (P+l loop); SD9:417–438; SD10:439–461; SD11:462–480; SD12:481–498. The alignment of functional subdomains for protein kinase genes was done using structure-informed multiple sequence alignment.

**Figure 2:**
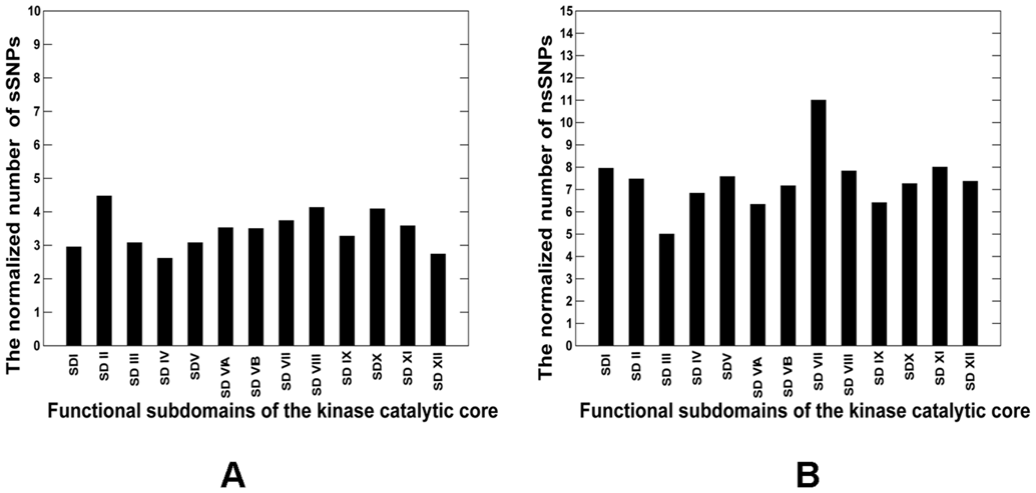
The Distribution of SNPs Types across Functional Subdomains of the Kinase Catalytic Core. The distribution of kinase sSNPs is shown in panel (A) and the distribution of sSNPs is presented in panel (B).

The integration and mapping of diverse data types in CKMD provided a convenient framework for kinome-wide analysis of sequence-based and structure-based signatures of cancer mutations. Genetic variations in protein kinase genes are widely spread across both phylogenetic and structural space, and only a subset of all SNPs could be directly mapped to the kinase catalytic domain. We began by analyzing the distribution of various SNPs categories that could be mapped onto the 12 functional subdomains (SDs) of the kinase catalytic core [**7**] (**Figure 1**). Structural mapping of sSNPs resulted in a uniform coverage of kinase subdomains, showing only a weak preference towards SD II which has no obvious functional role in kinase regulation (**Figure 2A**). In contrast, the distribution of nsSNPs highlighted the preferential bias towards specific functional regions. Indeed, functionally important P-loop (SD I), hinge region (SD V), catalytic loop (SD VIB), and especially activation loop (SD VII) along with the downstream P+1 loop region (SD VIII) tend to be more densely populated (**Figure 2B**). The P+1 segment links the subdomains in the C-terminal lobe with the ATP and substrate binding regions in the N-terminal lobe. Moreover, the P+1 loop is directly connected to the F-helix, which serves as a central scaffold in the assembly of active kinase form [**85]**–[**87**].

**Figure 3:**
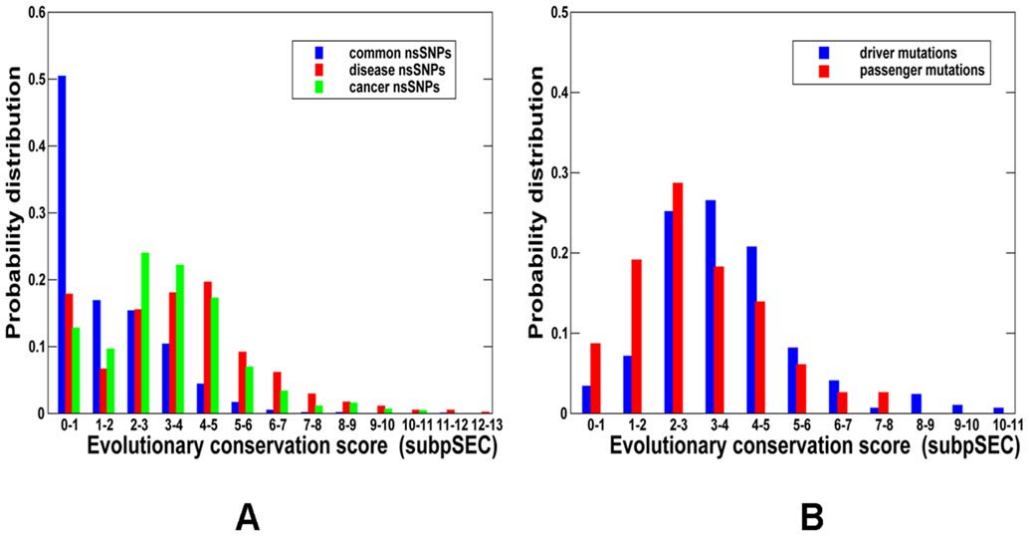
The Distribution of nsSNPs Types across Evolutionary Conservation Levels. (A) The probability distribution of common nsSNPs (shown in blue bars), disease-causing SNPs (shown in red bars) and cancer-causing nsSNPs (shown in green bars) as a function of evolutionary conservation level. (B) The probability distribution of cancer driver mutations (shown in blue bars) and passenger nsSNPs ( shown in red bars) as a function of evolutionary conservation level. For both panels (A) and (B), a higher score corresponds to a higher level of conservation.

The kinase catalytic domain harbors a significant number of nsSNPs falling into three major categories: common and likely neutral nsSNPs, inherited disease-causing nsSNPs, and cancer-causing (somatic) nsSNPs. We analyzed evolutionary conservation patterns among these three different categories of kinase nsSNPs (**Figure 3**). A measure of conservation was derived from the absolute value of the substitution position-specific evolutionary conservation score, termed “subPSEC,” which was obtained by aligning a given protein against a library of Hidden Markov Models (HMM) representing distinct protein families [**88]**, [**89**]. The score was defined as -|ln(P_aij_/P_bij_)|, where P_aij_ is the probability of observing amino acid a at position i in HMM j. According to the PANTHER website [**89**], a score of -3 would correspond to an estimated 50% probability that the SNP may be a disease causing variant. The SNPs conservation profiles for kinase genes could be described as the absolute value of subPSEC score, where the higher the score, the greater the degree of evolutionary conservation. The distribution of common nsSNPs was biased towards a lower level of conservation, as would be expected for neutral variants with little or no functional significance. Cancer-associated nsSNPs appeared to fall into positions with a higher level of conservation than common nsSNPs, yet could be as conserved as disease-causing nsSNPs (**Figure 3A**). This analysis indicated that either cancer-associated nsSNPs may not necessarily fall into evolutionary highly conserved positions, or the distribution may be skewed towards a lower conservation level by cancer variants of no functional consequence (passenger mutations). Using a recently developed SVM-based method capable of predicting functionally important cancer mutations [**70]**, [**71**], we compared the evolutionary conservation distributions of cancer driver mutations and passenger mutations at different levels of conservation (**Figure 3B**). Although the predicted cancer driver mutations did fall at the positions exhibiting slightly higher conservation level, as compared to the passenger mutations, the difference was rather modest. Hence, it appeared that cancer mutations in protein kinases may not display strong sequence conservation signals and consequently, functional importance of kinase genetic variants may not be directly related with their evolutionary conservation.

**Figure 4:**
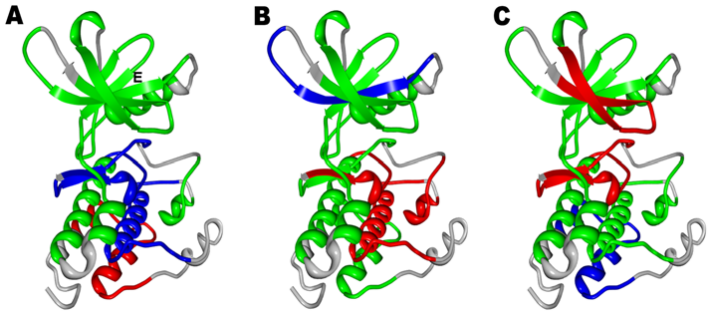
Structural Mapping of nsSNPs onto the Kinase Catalytic Domain. Structural mapping is shown for common nsSNPs (A), disease-causing nsSNPs (B), and cancer-causing nsSNPs (C). In all panels the green coloration represents regions with a SNP frequency equivalent to what would be expected by random chance, blue coloration represents regions that are statistically devoid of SNPs, and red coloration depicts regions that are statistically enriched in SNPs. Enrichment of SNPs in these regions was calculated as described in the Materials and Methods section. For clarity, the SNPs density was mapped onto a representative kinase crystal structure (EGFR, pdb entry 1M14) by projecting the multiple sequence kinase alignment onto the protein structure.

**Figure 5:**
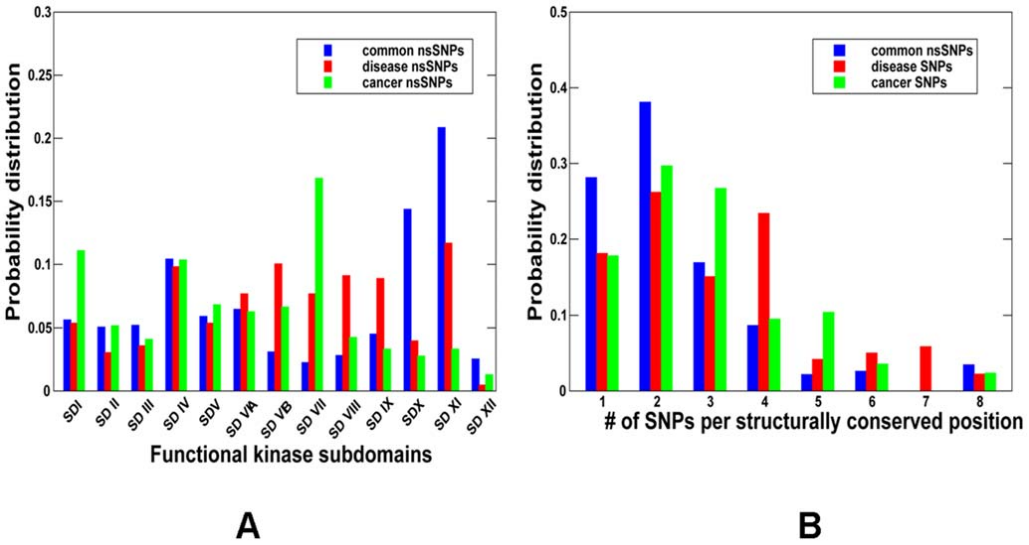
The Distribution of nsSNPs Types across Functional Subdomains of the Catalytic Core. (A) The distribution of common nsSNPs (shown in blue bars), disease-causing nsSNPs (shown in red bars), and cancer-causing nsSNPs (shown in green bars) in the functional subdomains of the kinase catalytic core. The expected probability of a SNP occurring in a kinase subdomain region was calculated for each SNP type as described in the Materials and Methods section. (B) The position-specific distribution of common nsSNPs (shown in blue bars), disease-causing nsSNPs (shown in red bars), and cancer-associated nsSNPs (shown in green bars) across different categories of structurally conserved mutational hotspots as determined by the number of SNPs per structurally identical position.

We also analyzed molecular determinants of genetic variations in protein kinases utilizing CKMD resource for a comprehensive structural mapping of nsSNPs onto the kinase catalytic core. The database-driven analysis revealed a differential enrichment of SNPs categories in functional regions of the kinase domain (**Figures 4**, **5**). Common nsSNPs tend to be randomly distributed within the catalytic core, only sparsely populating functional segments of the catalytic core, such as the catalytic or activation loops, whereas these nsSNPs more densely occupy evolutionary unconserved regions of the C-terminal tail (**Figure 4A**). The disease-causing nsSNPs primarily mapped to the regions involved in regulation and substrate binding, such as the APE-loop and the P+1 region, as well as the catalytic loop (**Figure 4B**). Cancer-associated nsSNPs tend to target regions directly involved in the catalytic activity that are mainly localized in the P-loop, activation loop and catalytic loop (**Figures 4C**). The distribution of kinase nsSNPs across functional kinase subdomains reinforced the notion that the kinase regions that are enriched (or devoid) of SNPs could be markedly different across the three mutation types, with a minimal overlap. Indeed, the distribution shows a clear preference for cancer-causing nsSNPs to accumulate mostly in the activation loop region (SDVII) as well as populating the P-loop (SD I) (**Figure 5A**). A significant number of disease-associated nsSNPs were not directly involved in the ATP binding, but rather buried in the catalytic core. Interestingly, the P+1 loop and the residues that anchor this pocket to the F-helix were some of the most enriched in disease-associated mutations, but not cancer-causing mutations. These results corroborate with previous findings indicating that disease-associated mutations could primarily affect the kinase regions involved in functional regulation, allosteric interactions and substrate binding [**72**].

Functional differences across different mutation types could be also reflected in the position-specific distribution of nsSNPs at the mutational hotspots determined by the number of structurally equivalent protein kinase positions (**Figure 5B**). The distribution of common nsSNPs, that have little or no functional affect and could be randomly distributed throughout the catalytic core, was dominated by weakly conserved positions mutated in a single, or two protein kinases. In contrast, the disease-causing nsSNPs tend to be concentrated at structurally equivalent positions, with a significant excess of mutations occurring at positions mutated in four or more different protein kinases. The position-specific distribution of cancer nsSNPs was shifted towards a higher number of nsSNPs per position, probably due to the selection of tumorigenic mutational hotspots shared across multiple protein kinases (**Figure 5B**).

### Structural Bioinformatics Analysis of Kinase Mutational Hotspots

**Figure 6:**
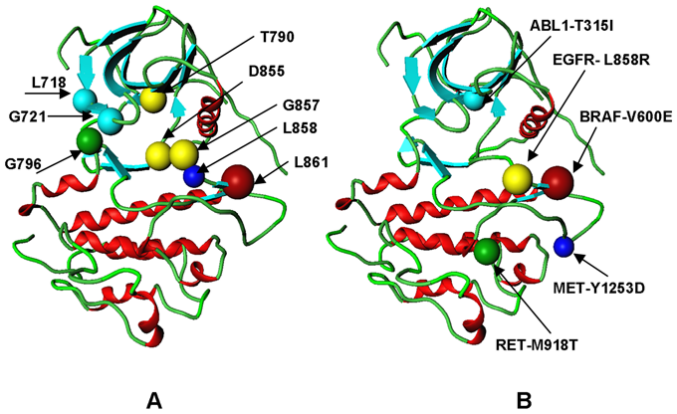
Structurally Conserved Mutational and Oncogenic Hotspots in the Kinase Catalytic Domain. (A) Structural localization of the conserved mutational hotspots is illustrated using the crystal structure of the active EGFR kinase (pdb entry 2J6M). The large-size red ball corresponds to the structural position of L861, and denotes localization of the largest mutational hotspot shared in 8 different kinases. The medium-size yellow balls correspond to structural positions of T790, D855, and G857 residues (respective mutational hotspots shared by 6 different kinases). The smaller green ball corresponds to G796 position (5 structurally conserved kinase mutations); the cyan balls correspond to L718 and G721 positions (each position denote residues with 4 cancer mutations); and the smallest blue ball corresponds to L858 position (3 structurally conserved kinase mutations). Cancer mutation hotspots in protein kinases are largely localized within the P-loop, hinge region, and activation loop. See also Table S1 for a comprehensive annotation of structurally conserved mutational hotspots. (B) Structural localization of cancer driver mutations with the high oncogenic potential is illustrated using the crystal structure of the active EGFR kinase (pdb entry 2J6M). The dominant oncogenic mutations are BRAF-V600E, KIT-D816V, and PDGFRa-D842V which all correspond to the same structurally conserved mutational hotspot. Structural annotation of cancer driver mutations is arranged according to their oncogenic potential as determined by the frequency of observing respective somatic mutations in the protein kinases genes. The higher the oncogenic potential of the cancer drive, the larger the ball denoting structural position of the respective mutation.

Kinome-wide analysis of sequence and structure-based signatures of cancer mutations, revealed that a significant number of cancer mutations could fall at structurally equivalent positions within the catalytic core. These structurally conserved mutations tend to cluster into specific mutational hotspots which may be shared by multiple kinase genes. Cancer mutation hotspots in protein kinases are largely localized within the P-loop, hinge region, and activation loop (**Figure 6A**, **Table S1**). Of special interest is a spectrum of EGFR, ABL, MET, FLT3 and KIT cancer mutations that correspond to the same structurally conserved position in the activation loop, which appeared to be mutated in at least 8 different kinases (**Figure 6A**, **Table S1**). This site corresponds to the known driver mutations BRAF-V600, FLT3-D835, KIT-D816, PDGFRa-D842, MET-D1228, EGFR-L861, ABL-L387, and ErbB2-L869. Despite a sequence-specific conservation pattern, many mutations at this structurally conserved position are commonly occurring activating mutations, including D1228H/N/V in MET [**90]**, [**91**], D835E/F/H/N/V/Y in FLT3 [**92]**, [**93**], D816E/F/H/N/I/V/Y in KIT [**94]**, [**95**] and V600D/E/G/K/L/M/R in BRAF [**96**]. In some cases, these mutations could have important implications for targeted inhibitor therapies by leading to drug resistance effects in KIT [**97**], BRAF [**98**], EGFR [**99**], ABL [**100**], and MET [**101**]. Another functionally important mutational hotspot corresponds to the conserved gate-keeper kinase position and includes ABL-T315I, EGFR-T790M, KIT-T670E, and PDGFRα-T674I variants (**Figure 6A**, **Table S1**). Some of the structurally equivalent positions could be conserved across the kinome, as the aspartate and glycine residues from the DFG motif (corresponding to the reference positions EGFR-D855 and EGFR-G857), as well as a conserved glycine in the hinge region (which corresponds to the EGFR-G796 reference position). There are examples of cancer mutations displaying a subgroup level of conservation, including EGFR-L858 position, which bears a conserved leucine in EGFR and ABL kinases, or a conserved aspartate shared in FLT3, KIT, MET, PDGFRα.

While most of the cancer driver mutations are likely to be rather rare, it is striking that a significant number of functionally important cancer mutants fall at structurally conserved positions within the kinase catalytic core. Moreover, we have observed that structurally conserved hotspots of cancer driver mutations often bear mutations with a high oncogenic activity (**Figure 6B**). A quantitative characterization of “oncogenicity” could be described in a variety of ways, including cell transformation potential, substrate utilization, and catalytic efficiency. However such data are typically available only for a limited number of genes and mutations and are not suitable for genome-wide analysis. We used a convenient definition of an oncogenic potential that may be offered by using the frequency profiles of somatic mutations in the protein kinases genes obtained from the COSMIC repository [**82**]. This analysis revealed that a rather small number of somatic kinase mutations with the known oncogenic potential could emerge with a high frequency in the mutational samples (**Table S2**). Strikingly, these functionally important mutations fall into major structurally conserved positions in the kinase catalytic domain. Indeed, highly oncogenic mutations BRAF-V600E, KIT-D816V, and PDGFRa-D842V belong to the largest mutational hotspot (**Figure 6B**). The functional importance of oncogenic kinase mutations from mutational hotspots such as ABL-T315I, EGFR-L858R, and RET-M918T, is also widely recognized. For instance, structurally conserved RET-M918T and MET-M1250T cancer drivers are situated in the substrate binding C-lobe of the kinase core (**Figure 6B**) and are known to be associated with oncogenic activation by displaying the highest transforming potential among known RET [**102]**–[**106**] and MET mutations [**107]**–[**110**]. The presented analysis suggests that structurally conserved hotspots in the kinase catalytic domain may be statistically enriched by mutations with a high probability of being cancer drivers. We argue that the preferential structural localization of oncogenic mutations in the activation loop and the substrate binding C-lobe of the kinase domain may be determined by their strategic location critical for the kinase autoinhibition, regulation and allosteric interactions in signal transduction networks.

### Structural and Energetic Signatures of Kinase Mutational Hotspots

Structural modeling and energetic analysis of cancer mutation effects can provide further insights into molecular mechanisms of kinase activation. We employed homology modeling and MD simulations to analyze whether structurally conserved cancer drivers that target the same tumorigenic hotspot in the kinase catalytic domain may also share a common activation mechanism. Molecular modeling focused on a quantitative comparison of MET-D1228V, MET-D1228H [**90]**, [**91**], FLT3-D835V, FLT3-D835E [**92]**, [**93**], and KIT-D816V, KIT-D816H [**94]**, [**95**] mutants. Substitutions of D835 in FLT3 and D816 in KIT result in the constitutive activation of the receptor, this residue has been suggested to play an important regulatory role. The crystal structures of FLT3 [**111**], KIT [**112**] and MET kinases [**113]**, [**114**] have suggested that cancer mutations may destabilize the autoinhibited wild-type (WT) form. It is important to note that structural modeling studies were performed to evaluate the extent of local perturbations that could be induced by cancer mutations on the autoinhibited kinase structure. Given the absence of high resolution crystal structures of kinase cancer mutants and nature of large conformational changes caused by activating mutations, we focused on understanding local functional effects of cancer mutations rather than attempting to make computational predictions of the mutant structures.

**Figure 7:**
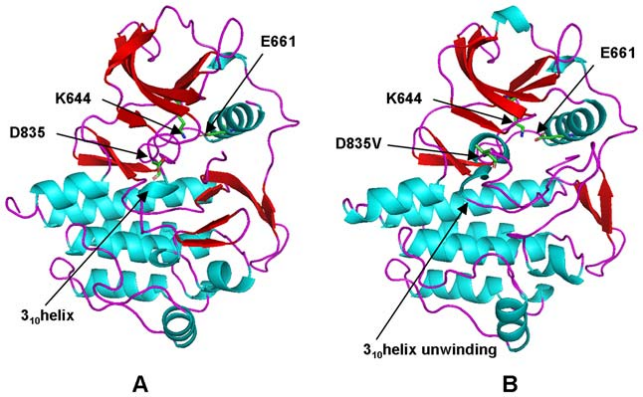
Structural Modeling of the FLT3-D835V Mutant. (A) The crystal structure of the autoinhibited wild-type FLT3 (pdb entry 1RJB). The position of D835 and key conserved residues K644 and E661 are highlighted. The location of the critical 3_10_-helix is indicated with an arrow. (B) Structural model of FLT3-D835V cancer mutant. Structural change in FLT3-D835V position and unwinding of the 3_10_-helix are highlighted with arrows.

**Figure 8:**
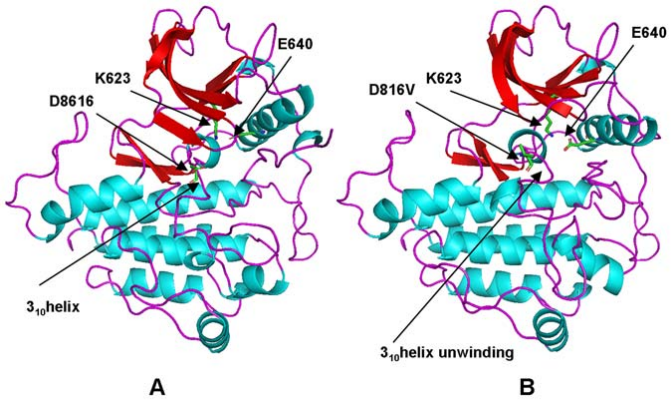
Structural Modeling of the KIT-D816V Mutant. (A) The crystal structure of the autoinhibited wild-type KIT (pdb entry 1T46). The position of D816 and key conserved residues K623 and E640 are highlighted. The location of the critical 3_10_-helix is indicated with an arrow. (B) Structural model of KIT-D816V cancer mutant. Structural change in KIT-D816V position and unwinding of the 3_10_-helix are highlighted with arrows.

**Figure 9:**
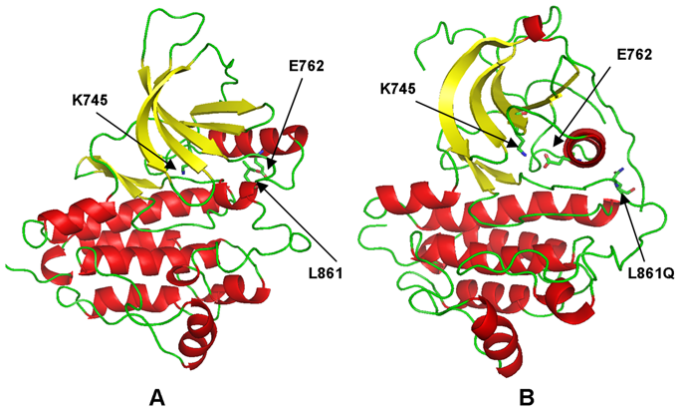
Structural Modeling of the EGFR-L861Q Mutant. (A) The inactive, Src-like structure of EGFR (pdb entry 2G7). The position of L861 is indicated with an arrow. The conserved salt bridge between K645 and E762 is broken in the inactive structure. (B) The model of the EGFR-L861Q mutant displays the active-like conformation of the activation loop. The new position of EGFR-L861Q residue and the restored salt bridge between K745 and E762 are indicated with arrows.

Homology modeling and MD simulations of commonly occurring activating mutations in this mutational hotspot revealed a significant local reorganization of the autoinhibited kinase conformation. This is reflected in the local structural variations near the site of mutation (root mean square deviations, RMSD = 3 Å−4 Å) (**Table S3**). The majority of cancer mutations resulted in moderate global changes, but considerable local structural changes near the mutational site and in the activation loop. The results revealed that structurally conserved FLT3-D835V (**Figure 7**) and KIT-D816V mutations (**Figure 8**) enhanced the local protein mobility near the mutational site and destabilized the autoinhibited kinase conformation through a similar molecular mechanism. Interestingly, FLT3-D835 and KIT-D816 participate in stabilization of the 3_10_-helix (**Figures 7A**, **8A**), which includes a stretch of residues (I836, M837, S838, D839, N841 in FLT3 and I817, K818, N819, D820 and S821 in KIT). During simulations the 3_10_-helix rapidly unfolded and remained in the unfolded state for both FLT3-D835V (**Figure 7B**) and KIT-D816 mutants (**Figure 8B**). Local perturbations induced by these mutations caused similar disruptions in the interaction networks responsible for stabilization of the inactive kinase form. In agreement with earlier studies [**115]**–[**117**], our results confirmed that deleterious effects of FLT3-D835V and KIT-D816V substitutions could primarily result from destabilization of the 3_10_-helix motif that is critical for the integrity of the inactive kinase form. Homology modeling and MD refinement of the EGFR-L861Q mutant, initiated from the inactive, Src-like EGFR crystal structure (**Figure 9A**), reproduced conformational changes in the activation loop leading to the active kinase form **Figure 9B**). This may be attributed to a considerable incompatibility of the activating mutation with the Src-like structure of the WT EGFR. While the hydrophobic Leu-861 is packed in a hydrophobic core of the WT structure (**Figure 9A**), switching to a polar residue triggered a conformational transition of the activation loop folding outwards, towards an active-like kinase state (**Figure 9B**).

**Figure 10:**
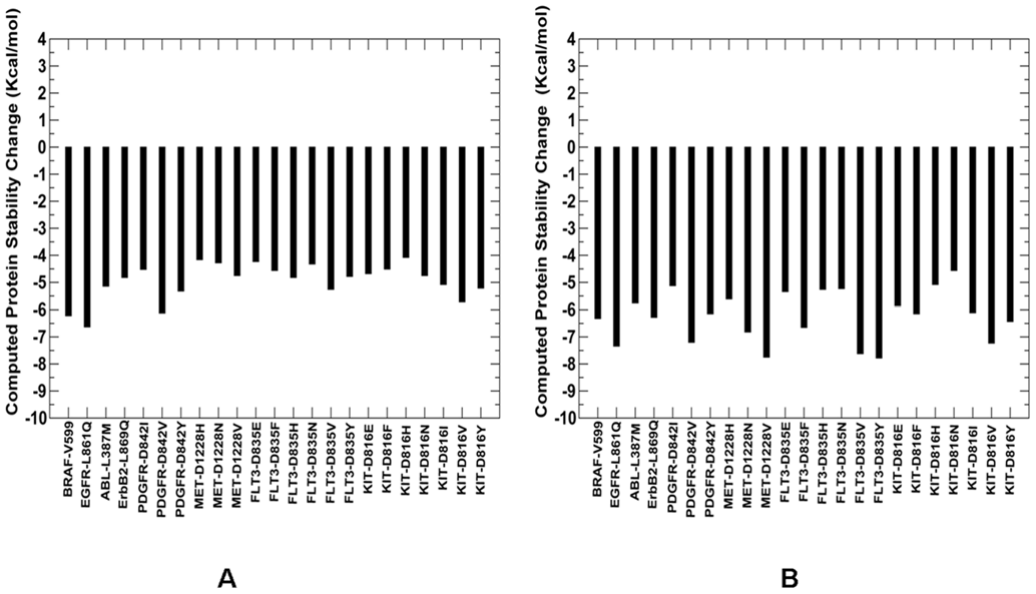
Protein Stability Analysis of the Cancer Mutation Hotspot. Protein stability differences calculated between the WT and mutants for structurally conserved mutations using CUPSAT (A) and FOLDx approaches (B). Negative values of protein stability changes correspond to destabilizing mutations.

According to our recent findings [**56]**, [**57**], cancer mutations in ABL and EGFR kinases, that display high oncogenic activity, may also induce the greater differential effect on thermodynamic stability of the inactive and active kinase forms. These energetic factors may serve as thermodynamic catalysts of kinase activation by cancer mutations. In line with this hypothesis, structural signatures of the cancer mutational hotspot may manifest in deleterious protein stability changes in the inactive state of the enzyme, thereby promoting transitions to the constitutively active kinase form. In the present study, we verified and expanded the initial conjecture by analyzing structural mapping of mutational hotspots and performing computational evaluation of protein stability changes using CUPSAT and FOLDx methods (**Figures 10** **,** **11**). Both approaches revealed a consistent trend, whereby commonly occurring activating mutations with an appreciable oncogenic activity resulted in a considerable destabilization of the autoinhibited WT structure (**Figure 10**). For example, mutations D1228H, D1228N, and D1228V in MET from the mutational hotspot are known to have significant oncogenic transformation effect of NIH 3T3 cells [**118]**, [**119**]. Accordingly, these mutations were shown to have a significant destabilization effect on the protein structure (**Figure 10**).

**Figure 11:**
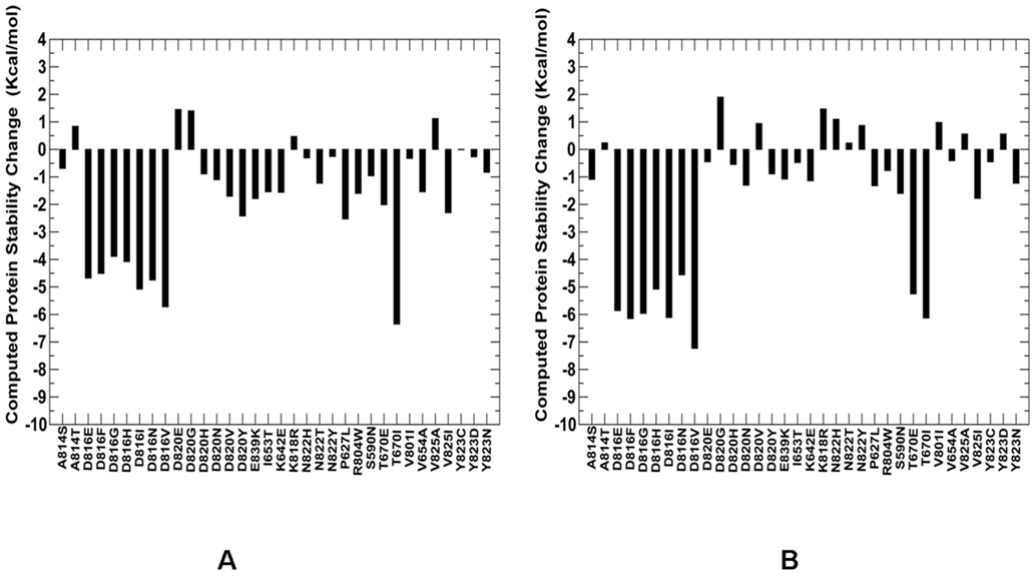
Protein Stability Analysis of KIT Mutations. Protein stability differences between the WT and mutants for a panel of KIT mutations using CUPSAT (A) and FOLDx approaches (B). The panel included both disease-causing mutations and commonly occurring cancer mutations at D816 position. Negative values of protein stability changes correspond to destabilizing mutations.

In order to illustrate functional significance of structural effects and concomitant protein stability changes for kinase cancer mutations, we compared protein stability differences between oncogenic KIT mutations at the D816 position and a spectrum of disease-causing KIT variants (**Figure 11**). A considerable destabilization effect on the autoinhibited inactive kinase was observed for the activating KIT mutations. In contrast, disease-causing SNPS only marginally affected protein stability of the WT KIT structure. Despite simplified energy models employed in the CUPSAT and FOLDx approaches, we observed consistent trends, capturing highly oncogenic mutations as the mutations which elicit larger and more detrimental protein stability changes. These results are consistent with our earlier studies; supporting the hypothesis that functional role of cancer mutations may be associated with their impact on the protein kinase stability.

## Discussion

Development of the integrated bioinformatics resource CKMD has enabled structure-based functional annotation and prediction of cancer mutation effects in protein kinases. Structural mapping of kinase genetic variants onto aligned crystal structures and mutational models has allowed to characterize molecular effects of nsSNPs. We have found an enrichment of different categories of SNPs in the different structural regions of the kinase domain, suggesting structure-based determinants responsible for selection of tumorigenic mutational hotspots. The distributions of nsSNPs types has shown that (a) neutral kinase nsSNPs are randomly distributed within the catalytic core; (b) disease-causing nsSNPs map to regulatory and substrate binding regions; and (c) cancer-causing nsSNPs can target catalytic and nucleotide binding functions, preferentially clustering in the activation loop of the kinase domain. Based on these results, we could speculate about potential diversity of structural mechanisms that may be associated with the effects of genetic alterations. It is possible that disease-causing mutations may function by perturbing the local environment near the organizing F-helix, which is responsible for maintaining structural plasticity and correct positioning of the key catalytic and regulatory spine regions [**85]**–[**87**]. On the other hand, structural effects of cancer-causing mutations may manifest in perturbing flexible regions that are directly involved in conformational transitions between inactive and active kinase forms. The preferential localization of cancer-causing mutations in the P-loop and the activation loop may lower the energetic barrier for triggering the dynamic imbalance shifted towards the constitutively active kinase conformation. The earlier analysis of protein kinase motions indicated that conformational motions in functionally important protein regions which harbor cancer mutations, namely the P-loop and activation loop, are coupled and may be highly correlated [**56]**, [**57**].

Although kinase cancer mutations may not exhibit a strong sequence conservation signal, we have identified a number of structurally equivalent positions within the protein kinase catalytic core can be frequent targets of tumorigenic mutations. These structurally conserved mutations tend to cluster into specific mutational hotspots which may be shared by multiple kinase genes. Sequence and structure-based methods were used to characterize molecular determinants of mutational hotspots in protein kinases. We have determined that structurally conserved hotspots in the kinase catalytic domain can be often enriched by cancer driver mutations with a high oncogenic potential. Structural modeling and energetic analysis of the mutational hotspots have also suggested a common molecular mechanism of kinase activation by cancer mutations, which may be determined by a combined effect of the partial destabilization of the inactive state and a concomitant stabilization of the active-like form of the enzyme. Furthermore, the results have indicated that cancer mutations with the higher oncogenic potential can have a greater differential effect on thermodynamic stability of the inactive and active kinase forms. Structure-based computational prediction and analysis of cancer mutation effects may thus be helpful for integrative cancer biology studies exploring the molecular pathology of tumorigenesis.

Ongoing development of database-oriented research tools within the CKMD environment will allow for automated structural and network-based bioinformatics analyses of rapidly growing knowledge-base of resequencing data on protein kinase genes. Further integration of genetic, functional, and structural insights about the molecular basis of tumorigenesis into robust bioinformatics infrastructure can ultimately help to discover molecular signatures of cancer mutations.

## Materials and Methods

### The Database Content and Organization

CKMD was developed as a bioinformatics resource for structure-functional analysis of genetic variations in protein kinases. We employed MySQL as a relational database management system for storing and managing the information content. Perl, a widely used scripting language was used to parse the data into various table forms. PHP5 Hypertext preprocessor was used in the design of the database interface, while Apache was used as the web server. Data stored in CKMD were mainly gathered from NCBI [**74]**–[**76**], COSMIC [**82**], SwissProt [**120]**–[**122**], and Protein Data Bank (PDB) [**123**]. We have also integrated non-redundant information about genetic variations in protein kinases from more specialized resources PupaSNP [**73**], KinMutBase [**77]**, [**78**], BTKbase [**79**], HGMD [**80]**, [**81**], PKR [**83**], and MoKCa [**84**].

Main entries in CKMD were indexed as genes and each gene entry contained many sub-entries of related information associated with that gene. We opted the gene id (GeneID) from Entrez Gene database as the unique identifier to index all entries in CKMD. This was partly due to the fact that the COSMIC database also referenced to GeneID in its entries. SwissProt, however, did not reference to GeneID and thus we developed a relation that matched SwissProt accession numbers with GeneIDs. This relation was crucial to coherently incorporate SwissProt data into CKMD along with the data from other sources. The raw data gathered from NCBI, SwissProt, and COSMIC were text files. All MySQL tables in CKMD referenced to either GeneID or SwissProt accession number. For each SNP entry, information about its position, nucleotide change and corresponding amino acid change was uniquely mapped on the protein kinase sequence and structure. The main information sources and a general architectural framework of CKMD are summarized in the design diagrams (**Figure S1**).

CKMD provides a simple and intuitive user interface that allows users to browse, search, download, and analyze genetic, sequence, structure and functional data on protein kinase data within a single integrated source. There are five main options available in CKMD: Composite, Browse, Search, Download, and Statistics. The “Composite” option offers a convenient and transparent way to view all information stored in CKMD for kinases genes. The “Browse” option allows to browse through entries in CKMD in three major categories: Gene, Mutation, and Structure. The “Search” option permits to query CKMD for a particular entry using many different searching criteria. The “Download” option allows to download and view all available protein kinase crystal structures and a large number of mutational models. Finally, the “Statistics” option offers various sequence and structure-based statistical analyses of SNPs distributions across kinase genes. The important CKMD functionality is that the database stores and provides a convenient access to protein kinase crystal structures and mutational models with the mapped nsSNPs. A total of 989 crystal structures corresponding to 126 kinase genes were collected from PDB and consolidated in CKMD. To facilitate structure-functional analysis of genetic variations in kinase genes, all crystal structures and mutational models were structurally aligned using a java-based multiple alignment tool STRAP (http://www.charite.de/bioinf/strap) and TM-align algorithm [**124**]. We have developed Java applet using Jmol, an open-source Java viewer for chemical structures in 3D (http://www.jmol.org/), to provide graphical representation of protein kinase structures. This interface could allow users to load and view multiple and aligned protein kinase structures along with convenient tools for manipulation of three-dimensional structures, localization and molecular analysis of SNPs.

Protein kinase sequences were obtained from Kinbase (http://kinase.com/kinbase/). Common SNPs were retrieved from PupaSNP [**73**] and dbSNP [**74**] using the Ensembl data mining tool, Biomart (http://www.ensembl.org/Homo_sapiens/martview). The disease causing SNPs were retrieved from OMIM [**75]**, [**76**], KinMutBase [**77]**, [**78**], and HGMD resources [**80]**, [**81**]. Currently, there are 518 kinase gene entries in CKMD, both referenced in NCBI [**74]**–[**76**] and SwissProt database [**120]**–[**122**], and 7955 unique SNP entries corresponding to these kinase genes that are referenced in NCBI. These unique SNP entries include 3722 synonymous, 3985 missense, 75 nonsense and 173 frameshift mutations. We have also gathered 780 OMIM variant entries from NCBI and 3542 SwissProt variant entries. Cancer mutations were retrieved from OMIM [**75]**, [**76**] and COSMIC resources [**82**]. The complete lists of mRNA and protein products for each unique SNP entry were also included and cross-linked to NCBI database. All nsSNPs were assigned to positions in Kinbase protein sequence using flanking sequences in the Ensembl and Entrez Gene sequences because of higher confidence in Kinbase sequences versus other publicly available sequences. Corresponding positions in DNA sequences were determined using a combination of flanking sequences given in dbSNP data and Genewise (http://www.ebi.ac.uk/Wise2/).

### Motif-based and Structure-based Multiple Sequence Alignments

Motif-based alignments of kinase sequences to the catalytic core were first generated by implementation of the Gibbs motif sampling method [**125]**, [**126**]. This method identifies characteristic motifs for each individual subdomain of the kinase catalytic core, which are then used to generate high-confidence motif-based Markov chain Monte Carlo multiple alignments based on these motifs [**127]**, [**128**]. These subdomains define the core structural components of the protein kinase catalytic core. Intervening regions between these subdomains were not aligned. The nsSNPs were then mapped to the kinase catalytic domain in accordance with this alignment. Cancer driver predictions were performed by using the SVM approach as described in our earlier work [**70]**, [71****]. Sequence analysis was done with the aid of the subPSEC conservation measure [**88]**, [**89**].

To further verify structural distribution of nsSNPs in functional kinase regions, we also performed structure-informed multiple alignment of kinase sequences using PROMALS3D approach [**129**]. In this approach, 30 different kinase crystal structures (**Table S4**) (the maximum allowed limit of structural information used by PROMALS3D) and kinase catalytic domain sequences for 445 different genes were used for the multiple sequence alignment. The obtained alignment was then matched against the alignment of the kinase sequences with the available crystal structure to ascertain the quality of the sequence alignment. The predicted and observed residue ranges for the catalytic loop, hinge region, αC-helix, activation loop and P-loop are in excellent agreement with the observed residue ranges for these functional kinase regions **(Table S5**).

### Kinase SNP Distribution and Enrichment Analysis

Functionally important subdomains of the kinase catalytic core, as in the nomenclature defined by Hanks and Hunter [**7**], were examined to determine the distribution of nsSNPs and identify structurally conserved hotspots of functionally important mutations. The number of SNPs in each of the subdomains was calculated from the structure-informed multiple sequence alignment described in the previous section. The expected probability E(p) of a SNP occurring in a kinase subdomain region was calculated separately for each SNP type as previously documented [**71]**, [**72**]. In brief, the average length of each region was calculated as the weighted average of the region length in each kinase considered, where weights correspond to the total number of SNPs occurring within each kinase. This weighting helps avoid biases that might arise as a result of some kinases simply harboring more SNPs than others. The probability of a SNP occurring within a particular region purely by chance was computed as its weighted average length over the sum of every region's weighted average length . The probability (p-value) of the observed total number (*x*) of SNPs occurring within each region, where *n* is the total number of SNPs considered, was calculated using the general binomial distribution as follows:

If *x*/*n* < *E(p)*:
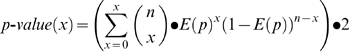



If *x*/*n* > *E(p)*:
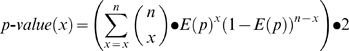



### Structural Modeling and MD Refinement of Kinase Cancer Mutants

We have also consolidated in CKMD all publically available crystal structures of WT and mutant protein kinases from PDB. A total of 989 kinase crystal structures corresponding to 126 genes were deposited in CKMD. Although a number of kinase crystal structures including mutants have been solved, there is still very little structural information about most cancer kinase mutants. To facilitate structure-functional analysis of cancer mutation effects in protein kinases we have generated and stored in CKMD structural models of a large number of protein kinase mutants (**Figure S2**). Only a subset of all SNPs can be directly mapped onto the kinase crystal structures. As a result, there are some protein kinases with the known WT crystal structure and known SNPs, yet no mutational models could be generated, because either all known mutations reside outside of the resolved crystal structure of the kinase catalytic domain or only synonymous mutations were available.

Structural modeling of nsSNPs was carried out using MODELLER [**130]**, [**131**] with a subsequent refinement of side-chains by the SCRWL3 program [**132**]. Initial models were built in MODELLER using a flexible sphere of 5 Å around mutated residue and the inactive crystal structures of the WT EGFR, FLT3, and KIT kinases as the templates. A protocol involving a conjugate gradient (CG) minimization, followed by simulated annealing refinement was repeated 20 times to generate 100 initial models for each studied mutant. In the optimization stage, we initially used 5000 steps of conjugate gradient (CG) minimization to remove unfavorable contacts and ensure sufficient relaxation of the local environment near mutational site. The predicted mutational models were chosen out of the 100 models as scored by the MODELLER default scoring function. These final models were then refined in 2ns MD simulations using NAMD 2.6 [**133**] with the CHARMM27 force field [**134]**, [**135**]and the explicit TIP3P water model as implemented in NAMD 2.6 [**136**]. Equilibration was done in stages by gradually increasing the system temperature in steps of 20K starting from 10K until 310K. At each stage, 10,000 equilibration steps was employed, while applying a harmonic restraining force of 10 Kcalmol^−1^Å^−2^ to all backbone Cα atoms. Subsequently, the system was equilibrated for 150,000 steps at 310K (NVT) and then for additional 150,000 steps at 310K using Langevin piston (NPT) to maintain the pressure. Finally the restrains were removed and the system was equilibrated for 500,000 steps to prepare the system for simulation. An NPT simulation was run on the equilibrated structure keeping the temperature at 310K and pressure at 1 bar using Langevin piston coupling algorithm. Nonbonded van der Waals interactions were treated by using a switching function at 10A and reaching zero at 12 Å distance.

### Protein Stability Calculations

To quantify the destabilization effect of cancer mutations on the inactive, autoinhibited kinase form, we computed the protein stability change upon these mutations using CUPSAT (Cologne University Protein Stability Analysis Tool) approach for the prediction and analysis of protein stability changes upon point mutations [**137]**, [**138**] and Foldx method [**139]**, [**140**]. In the CUPSAT approach, coarse-grained atom potentials and torsion angle potentials are used to predict protein stability upon point mutations. Foldx analysis of protein stability is based on the empirical force field which was developed for the rapid evaluation of the effect of mutations on the stability, folding and dynamics of proteins and nucleic acids. The free energy of folding is evaluated in this approach from the difference in Gibbs free energy between the crystal structure of the protein and a hypothetical unfolded reference state of which no structural details are known.

## Supporting Information

Figure S1 CKMD Architecture and Information Sources.
(0.20 MB TIF)

Figure S2 The Gene-based Distribution of Structurally Mapped Kinase Cancer Mutations. For clarity of presentation, only top 70 kinase genes that have cancer-causing nsSNPs mapped onto three-dimensional protein structure are presented.
(0.75 MB TIF)

Table S1 Structurally conserved cancer mutation hotspots in the protein kinase genes.
(0.03 MB XLS)

Table S2 Distribution of kinase cancer mutations in the mutational samples
(0.18 MB DOC)

Table S3 Analysis of the crystal structures and mutational models
(0.09 MB DOC)

Table S4 The list of protein kinase crystal structures used for the multiple sequence alignment by PROMALS3D.
(0.06 MB DOC)

Table S5 Structure-based multiple alignment of kinase sequences.
(0.04 MB XLS)
