# Supplementary figures and images for: Sequence and Structure Signatures of Cancer Mutation Hotspots in Protein Kinases
Source: PLoS One. 2009 Oct 16;4(10):e7485. doi: 10.1371/journal.pone.0007485 (PMC2759519; doi:10.1371/journal.pone.0007485)

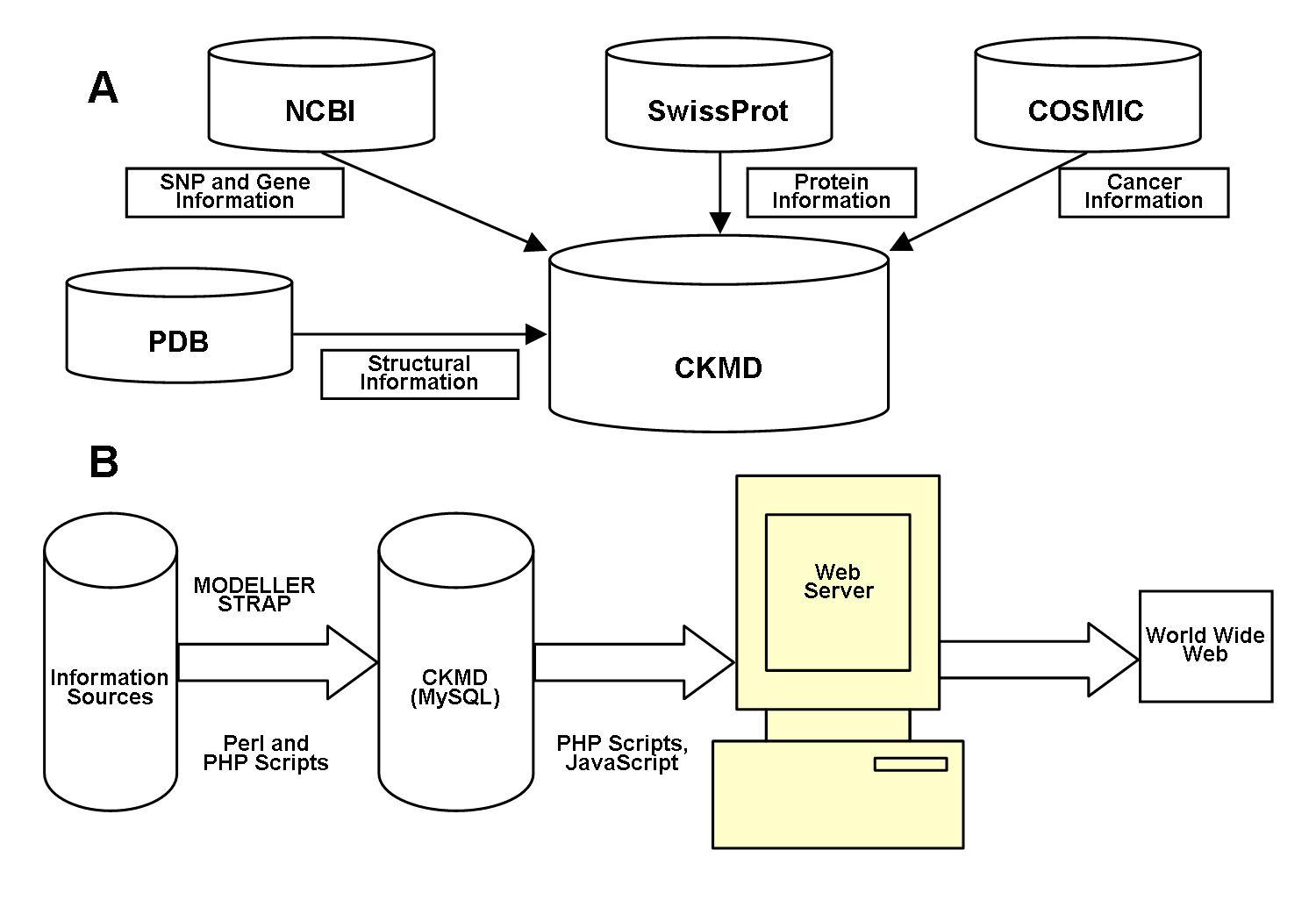

Supplement: Figure S1 — CKMD Architecture and Information Sources. (0.20 MB TIF) [file pone.0007485.s001.tif]

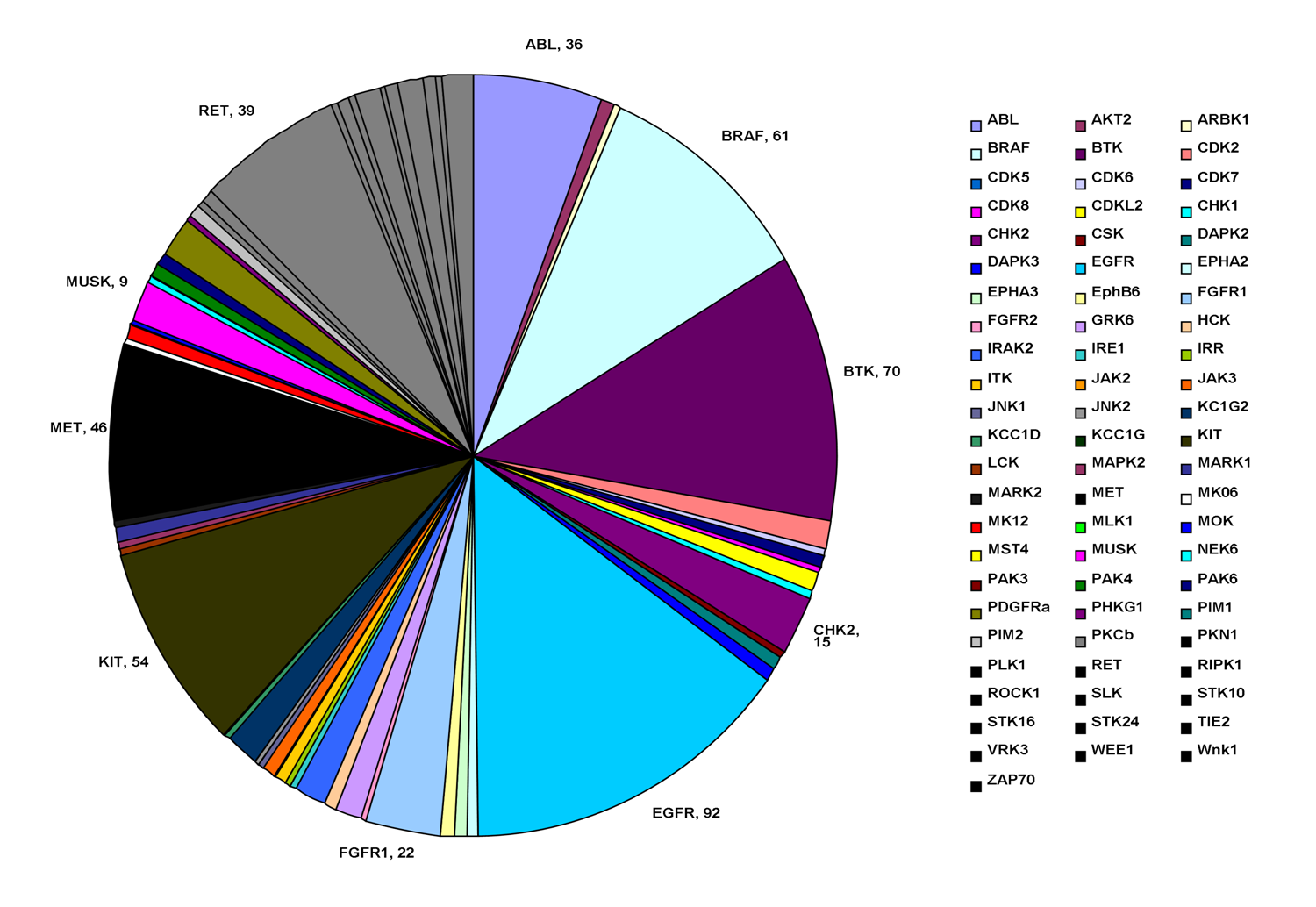

Supplement: Figure S2 — The Gene-based Distribution of Structurally Mapped Kinase Cancer Mutations. For clarity of presentation, only top 70 kinase genes that have cancer-causing nsSNPs mapped onto three-dimensional protein structure are presented. (0.75 MB TIF) [file pone.0007485.s002.tif]
