# Supplementary material for: Sequence and Structure Signatures of Cancer Mutation Hotspots in Protein Kinases
Source: PLoS One. 2009 Oct 16;4(10):e7485. doi: 10.1371/journal.pone.0007485 (PMC2759519; doi:10.1371/journal.pone.0007485)
Supplement: Table S2 — Distribution of kinase cancer mutations in the mutational samples (0.18 MB DOC) [file pone.0007485.s004.doc]

**Table S2. Distribution of kinase cancer mutations in the mutational samples***

| **Kinase Gene** | **Mutation** | **Mutational frequency** |
| --- | --- | --- |
| BRAF | V600E | 10406 |
| EGFR | L858R | 1828 |
| FGFR3 | S249C | 955 |
| KIT | D816V | 571 |
| FGFR3 | Y373C | 296 |
| RET | M918T | 243 |
| PDGFRA | D842V | 234 |
| FGFR3 | R248C | 187 |
| BRAF | V600K | 113 |
| EGFR | T790M | 105 |
| KIT | V559D | 91 |
| KIT | V560D | 84 |
| FGFR3 | G370C | 82 |
| KIT | N822K | 66 |
| KIT | V654A | 59 |
| EGFR | L861Q | 50 |
| KIT | L576P | 47 |
| KIT | D816H | 46 |
| KIT | D816Y | 46 |
| KIT | W557R | 44 |
| FGFR3 | G697C | 44 |
| FGFR3 | S371C | 42 |
| KIT | K642E | 38 |
| FGFR3 | K650E | 38 |
| EGFR | G719A | 38 |
| FGFR3 | K650M | 35 |
| ALK | F1174L | 34 |
| FGFR3 | A391E | 34 |
| EGFR | S768I | 33 |
| BRAF | K601E | 32 |
| KIT | V559A | 31 |
| KIT | W557G | 30 |
| EGFR | G719S | 30 |
| EGFR | G719C | 29 |
| BRAF | V600R | 28 |
| PDGFRA | V561D | 28 |
| KIT | V825A | 27 |
| KIT | V559G | 26 |
| MET | Y1253D | 26 |
| ALK | R1275Q | 26 |
| BRAF | V600M | 25 |
| BRAF | D594G | 24 |
| BRAF | V600A | 22 |
| KIT | Y823D | 21 |
| BRAF | G469A | 17 |
| KIT | V560G | 16 |
| KIT | M541L | 15 |
| KIT | T670I | 14 |
| BRAF | V600D | 14 |
| CSF1R | Y969C | 13 |

* Top 50 oncogenic kinase mutations ranked by their mutational frequency are shown. The frequencies of somatic mutations are obtained from COSMIC repository [**82]**.
