# Supplementary material for: Sequence and Structure Signatures of Cancer Mutation Hotspots in Protein Kinases
Source: PLoS One. 2009 Oct 16;4(10):e7485. doi: 10.1371/journal.pone.0007485 (PMC2759519; doi:10.1371/journal.pone.0007485)
Supplement: Table S3 — Analysis of the crystal structures and mutational models (0.09 MB DOC) [file pone.0007485.s005.doc]

**Table S3: Analysis of the crystal structures and mutational models**

| **Kinase Mutant** | **Global RMSD^#^** | **Local RMSD^*^** |
| --- | --- | --- |
| MET-D1228V | 1.241 | 4.202 |
| MET-D1228H | 1.588 | 3.968 |
| EGFR-L861Q | 6.264 | 5.743 |
| KIT-D816V | 1.405 | 3.866 |
| KIT-D816E | 0.700 | 3.588 |
| KIT-D816F | 0.779 | 3.075 |
| KIT-D816H | 0.864 | 3.887 |
| FLT3-D835E | 0.772 | 3.402 |
| FLT3-D835V | 1.436 | 3.032 |
| FLT3-D835N | 0.770 | 3.717 |
| FLT3-D835F | 0.821 | 2.933 |

^#^ Global RMSD denotes RMSD calculated for all protein Cα atoms * Local RMSD denotes RMSD calculated by taking local environment of the mutated residue which includes neighboring residues within 5Å radius.
