# Supplementary material for: Sequence and Structure Signatures of Cancer Mutation Hotspots in Protein Kinases
Source: PLoS One. 2009 Oct 16;4(10):e7485. doi: 10.1371/journal.pone.0007485 (PMC2759519; doi:10.1371/journal.pone.0007485)
Supplement: Table S4 — The list of protein kinase crystal structures used for the multiple sequence alignment by PROMALS3D. (0.06 MB DOC) [file pone.0007485.s006.doc]

**Table S4. The list of protein kinase crystal structures used for the multiple sequence alignment by PROMALS3D.**

| Kinase Gene | PDB |
| --- | --- |
| ABL | 1IEP |
| AKT2 | 1GZK |
| ARBK1 | 1OMW |
| BRAF | 1UWH |
| BTK | 1K2P |
| CDK2 | 1FVV |
| CDK5 | 1UNG |
| CDK6 | 1BLX |
| CDK7 | 1UA2 |
| CHK1 | 1IA8 |
| CSK21 | 1JWH |
| DAPK2 | 2A2A |
| DAPK3 | 1YRP |
| EGFR | 2J6M |
| HCK | 1QCF |
| JNK1 | 2H96 |
| KCC1G | 2JAM |
| KIT | 1T45 |
| LOK | 2J7T |
| MAPK2 | 1KWP |
| MARK2 | 1ZMU |
| MET | 2G15 |
| PAK1 | 1YHV |
| PIM1 | 1XQZ |
| PKCB | 2I0E |
| RET | 2IVS |
| ROCK1 | 2ETR |
| STK6 | 1MQ4 |
| WEE1 | 1X8B |
| WNK1 | 1T4H |
